# Qualitative evaluation of primary care providers experiences of a training programme to offer brief behaviour change counselling on risk factors for non-communicable diseases in South Africa

**DOI:** 10.1186/s12875-015-0318-6

**Published:** 2015-08-19

**Authors:** Zelra Malan, Robert Mash, Katherine Everett-Murphy

**Affiliations:** Family Medicine and Primary Care, Stellenbosch University, Box 19063, Tygerberg, 7505 South Africa; Chronic Diseases Initiative in Africa (CDIA), Faculty of Health Sciences, University of Cape Town, P/Bag X 3, Observatory, Cape Town, 7935 South Africa

## Abstract

**Background:**

The global epidemic of non-communicable disease (NCDs) has been linked with four modifiable risky lifestyle behaviours, namely smoking, unhealthy diet, physical inactivity and alcohol abuse. Primary care providers (PCPs) can play an important role in changing patient’s risky behaviours. It is recommended that PCPs provide individual brief behaviour change counselling (BBCC) as part of everyday primary care. This study is part of a larger project that re-designed the current training for PCPs in South Africa, to offer a standardized approach to BBCC based on the 5 As and a guiding style. This article reports on a qualitative sub-study, which explored whether the training intervention changed PCPs perception of their confidence in their ability to offer BBCC, whether they believed that the new approach could overcome the barriers to implementation in clinical practice and be sustained, and their recommendations on future training and integration of BBCC into curricula and clinical practice.

**Methods:**

This was a qualitative study that used verbal feedback from participants at the beginning and end of the training course, and twelve individual in-depth interviews with participants once they had returned to their clinical practice.

**Results:**

Although PCP’s confidence in their ability to counselling improved, and some thought that time constraints could be overcome, they still reported that understaffing, lack of support from within the facility and poor continuity of care were barriers to counselling. However, the current organisational culture was not congruent with the patient-centred guiding style of BBCC. Training should be incorporated into undergraduate curricula of PCPs for both nurses and doctors, to ensure that counselling skills are embedded from the start. Existing PCPs should be offered training as part of continued professional development programmes.

**Conclusions:**

This study showed that although training changed PCPs perception of their ability to offer BBCC, and increased their confidence to overcome certain barriers to implementation, significant barriers remained. It is clear that to incorporate BBCC into everyday care, not only training, but also a whole systems approach is needed, that involves the patient, provider, and service organisation at different levels.

## Background

Non communicable diseases (NCDs) are the leading cause of death globally and are projected to increase by 15 % between 2010 and 2020 [1]. This increase is attributable to the increasing numbers of children and adults exposed to the risk factors of obesity, hypertension, diabetes and tobacco addiction that contribute to NCD’s as well as to the aging of populations. The main behavioural risks underlying these risk factors are an unhealthy diet, physical inactivity, harmful alcohol intake and tobacco smoking.

In low and middle income countries (LMIC) NCD’s disproportionally affect younger adults, there are fewer resources to tackle the problem, and services are often poorly prepared for chronic care [2]. In South Africa, NCD’s are not only a burden on the health care system, but are also putting increasing demands on patients, families and society. Patients and families must cope with the consequences of ill health and sometimes disabling complications, while the impact on working age adults and productivity makes this a developmental issue for government. There is also an emerging interaction between infectious and non-infectious chronic diseases. For example, inflammatory cardiac conditions are more common in HIV positive individuals and some side effects of anti-retroviral medication predispose patients to cardiovascular disease. The need for an integrated approach to chronic disease management based on universal principles is clear [2].

The National Department of Health (NDOH) has listed the control of NCDs as a key priority in their Strategic Plan [3]. Primary care providers (PCPs) are well positioned to address the challenges of both prevention and management of NCDs [4]. Preventing the common modifiable risk factors associated with NCDs is regarded as an important strategy. In line with World Health Organization (WHO) guidelines, brief behaviour change counselling (BBCC) in primary care is recommended for all four risky behaviours [4–6]. BBCC is defined as a short intervention of 3–5 minutes, usually delivered opportunistically as part of the normal consultation, and which ultimately aims to strengthen their beliefs about their own ability to change risky behaviours [7]. A patient centred approach is essential in assisting a patient to self-manage their chronic conditions and associated risk factors [6, 8].

Delivering BBCC in a patient centred way requires a significant change from the current model that is more orientated towards public health goals, vertical disease programmes and acute care. Patient-centred care requires an understanding of the individual’s complexity and context, and integration of programmatic and disease orientated guidelines with the patient’s unique situation and goals [9]. Care for chronic conditions is inherently different from care for acute conditions, as it requires a greater level of organisation that must be sustained over a patient’s lifetime, and higher levels of co-ordination between different health workers who may be involved [4].

Local research has shown that PCPs are sceptical about their ability to help patients change behaviour, partly because current training programmes in the Western Cape are not designed to build such capacity. Apart from inadequate training in counselling skills, they also report numerous other barriers such as language, lack of time, poor knowledge of lifestyle modification and poor continuity of care [10, 11].

This article reports on a study, which formed part of a larger research project that developed, implemented and evaluated a training intervention for PCPs on BBCC, and assessed providers’ competency in delivering a brief counselling intervention. The evidence-based 8-hour programme was designed to train participants in the 5 A’s best practice clinical guideline for BBCC [ask, alert, assess, assist, arrange] [12]. Although the 5 As provide a straightforward and easily remembered step-wise structure, the original guidelines do not emphasise the importance of a patient centred approach. In this study the 5 As were combined with the guiding principles of motivational interviewing (MI), which is a more flexible approach that is well suited to support self-management and evoke behaviour change [6, 8]. MI works by enhancing the patient’s own motivation to change and resolving their ambivalence.

MI has wide applicability in behaviour change conversations within health care settings, and is fundamentally different from the traditional directive and confrontational approaches used in everyday clinical practice. Traditionally, PCPs embody the role of the expert advice giver, and try to convince the patient why, what and how they should change. In MI the argument for change is evoked from the patient, expertly guided by the PCP, through a shared decision making process. The patient is recognised as the expert in their own life and the PCP values and respects their autonomy about how, when and what needs to change. The patient plays an active role, whilst the PCP provides structured direction, expert information and negotiates change sensitively, as an expert guide [6].

The results of the larger study suggested that training clinical nurse practitioners and primary care doctors in BBCC, in the South African context, was effective in the short term, and could result in significant adoption of the 5 As and a guiding style [13]. These results echoed international findings that have demonstrated that PCPs can be effective in providing BBCC. However, the local barriers to counselling in clinical practice remain unchanged. This article reports on a qualitative sub-study, which explored whether the training intervention changed participants attitudes and beliefs about BBCC once they had returned to their clinical practice after the training. The specific objectives of this study were therefore to explore:

1. If the training changed PCPs perception of their ability to offer BBCC
2. Whether they believed that the new approach could overcome the barriers to implementation in clinical practice and be sustained
3. The PCP’s recommendations on future training and integration of BBCC into curricula and clinical practice.

## Methods

### Study design

This was a qualitative study that used two data sources:

- Verbal feedback from participants at the beginning and end of the training course
- Individual in-depth interviews with participants once they had returned to their clinical practice

### Setting

The study was conducted within the context of the primary healthcare system of the Western Cape, where the vast majority of people make use of the public health care services and do not have access to specialised counsellors on lifestyle modification or behaviour change. Multi-morbidity is common and patients presenting with chronic diseases are managed by clinical nurse practitioners in either small clinics or larger health centres, and only referred to a primary care doctor occasionally [14]. Not all of these nurses are trained clinical nurse practitioners, and even clinical nurse practitioners receive only an additional one year of training to cope with the wide range of problems seen in primary care.

The training was developed as an 8-hour short course that combined theory; modelling and simulated practice with feedback, for both clinical nurse practitioners and primary care doctors in South Africa. The training programme combined the 5 As with a patient centred guiding style derived from motivational interviewing [Table 1]. The design, development, implementation and evaluation has been reported elsewhere [15, 16]. A training manual that summarised the model of BBCC and the underlying evidence as well as applying the model practically to each risk factor was distributed to each participant. In addition each participant received patient educational material on each risk factor that was designed to dovetail with the approach to BBCC. All of these printed materials can be accessed via the web at www.ichange4health.co.za.

**Table 1:** Summary of the training programme

Session	Time (minutes)	Purpose of session	Activities for session
1.1	15	Introductions and overview of programme and learning outcomes	Introduce the training programme in terms of the people involved, the intended learning outcomes and the process to be followed
1.2	30	Understand participant’s own prior experience of the challenges and successes of BBCC	Invite students to reflect in pairs and then share with the whole group on their prior experience with BBCC. This step was thought to be important in terms of building rapport between the trainers and participants, understanding the participant’s context, allowing them to express their ambivalence and frustrations and have these recognised, and helping to focus attention on behaviour change counselling.
1.3	45	Evidence for BBCC	Provide evidence of the current deficiencies in counselling, the reasons for them, the consequences for patients and health care providers.
			Provide evidence for the model of BBCC and its effectiveness.
			Allow time for discussion/questions.
1.4	30	Understand the guiding style	Identify the key characteristics of the guiding style by contrasting two DVD clips of BBCC – the one in a directing style and the other in a guiding style.
			Ask students to identify the key characteristics of each style, record and compare on newsprint.
2.1	40	Reflective listening	Talk: Give a brief overview of the theory of reflective listening
			Modelling: Demonstrate using DVD
			Practice: Using small group interactive exercises
2.2	40	Recognise, elicit and respond to change talk	Talk: Brief overview of theory from motivational interviewing
			Practical: Trainers reads out a list of statements and students drum on tables if they recognise change talk
2.3	40	Introduction to the 5 As	Talk: Overview of the 5 A steps, the purpose of each step and communication skills involved
			Allow time for discussion/questions
3.1	80	Applying the 5 As to each risk factor	● Form 4 groups
			● Each group looks at the training manual (5A steps and patient education material) for one behavioural risk factor
			● Form 4 new groups with one person from each of the previous groups
			● Each person teaches the others about their risk factor
			● Elicit feedback/discussion in whole group
3.2	40	Exchanging information	Talk: Brief overview of theory from motivational interviewing
			Modelling: Demonstrate elicit-provide-elicit with DVD
			Practice: Small group interactive exercises
4.1	30	Assess readiness to change	Talk: Brief overview of theory from motivational interviewing and application to the assess stage.
			Modelling: Demonstrate in role play or DVD
			Practice: Small group interactive exercises
4.2	60	Practice integrated BBCC	● Groups of 4
			● Allocate one different risk factor per person
			● Each person thinks of a patient to role play
			● Role play BBCC
			● Observe, give feedback and discuss
			● Facilitator to rotate to each group
4.3	25	Planning integration into real world	● Interview each other in pairs
			● Assess how ready your partner is to implement BBCC
			● Assist the person appropriately to plan change
			● Each person briefly gives feedback on their way forward to whole group
			● Discuss ways of ongoing learning with group
4.4	5	Closure and evaluation of workshop	Complete end of workshop with feedback form

### Study population and selection of participants

Our study population included primary care doctors and clinical nurse practitioners, from both the private and the public sectors. Participants were recruited by advertising the training as a short course offered by Stellenbosch University. During 2012–2013 three groups of nurses and three groups of primary care doctors completed the training programme. Twenty three nurses on the 1-year Diploma course at Stellenbosch University (Diploma in Clinical Nursing Science, Health Assessment, Treatment and Care), twelve family medicine registrars during their second year of training at the Universities of Stellenbosch or Cape Town, two general practitioners in private practice in Cape Town, and four family physicians working in the rural areas of the Western Cape were trained. In South Africa, doctors complete a 2-year internship (which includes 3-months of primary care) and 1-year of community service after graduation, and then they are free to enter private general practice, working as a general practitioner, or continue as a medical officer in the public sector with no further training. Since 2007 doctors can train as family physicians through becoming a registrar in an accredited 4-year postgraduate programme, and be registered as a specialist.

All of the participants were involved in the verbal feedback sessions at the beginning and end of the training. For the individual, face-to-face, in-depth interviews, six doctors and six nurses who had received the training were purposefully selected according to their ability to demonstrate BBCC at the end of training (2 with low scores, 2 with medium scores and 2 with high scores) in order to explore a range of experiences in applying the learning in clinical practice. Participant’s scores were obtained from quantitative measurement of their performance in delivering BBCC in audio recordings with standardised patients after training, using the MITI 3.1 tool, and has been reported elsewhere [13]. Logistical and budgetary limitations determined that these respondents be selected from the group of doctors and nurses, working in the Cape Town Metropole.

### Data collection

At the start of the training participants were asked to discuss in pairs their prior difficulties and successes with BBCC. These ideas and experiences were then shared with the group. At the end of the course these ideas were revisited and participants were asked to reflect on them again, in order to explore if there were any changes in their perceptions of BBCC. All these reflections were documented by the researcher in her field notes and on newsprint. All three authors were present for the group discussions during the training.

In depth interviews were conducted 2 weeks after the training at the primary care facilities where interviewees worked in the Cape Metropole. The researcher, who was a qualified family physician and spent many years working in private general practice, performed and audio-taped in-depth interviews in the interviewee’s choice of language, either Afrikaans or English. The researcher used an interview guide, and skills such as open ended questions, reflective listening, summarizing, and elaboration to conduct the interviews. The opening question used was “Could you tell me more about your feeling on brief behaviour change counselling before and after the training?” Topics that could then be discussed included their confidence to implement and sustain the new BBCC approach in their clinical practice, the pros and cons of this new approach, and their ideas on training other primary care providers.

### Data analysis

The researcher familiarised herself with the data collected during the training, by reading the field notes and newsprint and identifying the key ideas and grouping them into themes. Particular attention was given to how the themes changed from before to after training.

Interviews were transcribed verbatim and the transcripts were checked and corrected prior to analysis using Atlas-ti software [v.6.2.12 2011] and the framework method [17]. The framework approach to content analysis involves the following steps:

- Familiarisation: The researcher listened to the tapes, read the transcripts, and listed recurrent issues or ideas that emerged from the data.
- Construction of thematic framework: The researcher organised these issues and ideas into a framework that was aligned with the objectives of the study. All three authors validated the thematic framework. In Atlas-ti this related to a list of codes organised into families.
- Coding: The researcher applied the thematic framework systematically to all the data by annotating the transcripts with the codes using Atlas-ti.
- Charting: All the data from the specific codes included in a family in Atlas-ti were brought together in one document or chart.
- Mapping and interpretation: The researcher used the charts to interpret the data for themes and look for any associations or relationships between themes. The internal consistency of the interpretation was reviewed by all three authors.

Saturation was reached within the results, and therefore we did not include additional interviews. During the last interviews no new themes emerged, and therefore we did not feel it necessary to perform additional interviews.

### Ethical consideration

Informed consent was obtained from all participants. The confidentiality and privacy of all interviewees and participants were respected in data analysis and reporting, and no names were used. This study was approved by the Health Research Ethics Committees (HREC) at Stellenbosch University [Reference number: N11/11/321].

## Results

### Results from reflections before and immediately after training

Three training programmes were held for a total of 23 nurses and three training programmes for a total of 18 primary care doctors. Table 2 juxtaposes the interpretation of comments from the whole group at the beginning compared to the end of training regarding their approach to delivering BBCC. PCPs appreciated that there could be benefits for themselves and their patients if they shifted their counselling style from that of an authoritarian expert to more of an expert guide. Comments suggested an increase in confidence to provide counselling and the training appeared to enable a transition from a directing practitioner-centred style to a more guiding patient-centred style.

**Table 2:** Interpretation of the themes from field notes before and after training

Before training	After training
More authoritarian	More collaborative
They reported that patients do not listen to what nurses and doctors, as the experts, tell them to do. They felt that trying to change a patients mind to change a risky behaviour, was a difficult task.	They reported that they needed to listen more to hear what patients had to say, rather than telling them what to do. They reported that they recognised the need to change the way that they look at patients, and that incorporating a patient’s circumstances into a conversation about changing behaviour, was an important aspect of counselling.
More directing and ‘telling’ the patient what to do	More eliciting and strengthening the patient’s own reasons for change
They reported that patient’s don’t understand the importance of changing risky behaviour, and therefore needed to be educated about the importance of change.	They recognised that previously they were trying to change their patients by persuasion and argumentation, rather than simply helping patients to change for themselves according to their own reasons and in their own time.
Patients do not have control and choices about their behaviour	Respect patients control and choices
They felt responsible for their patient’s unhealthy behaviours, and reported that they found it challenging to counter a patient’s beliefs about not changing.	They reported feeling relieved when they understood why patients often do not change when they expected them to, and that they are not expected to argue about it, or feel frustrated, but rather to respect the patient’s choices.

### Results from individual interviews after return to clinical practice

Twelve individual interviews were conducted .The type of PCP and their study code (ID) is given after the quotations used below.

#### Attitudes and beliefs about BBCC

The PCPs who were interviewed reported feeling less frustrated about counselling in clinical practice, more confident in their ability to help patients and less sceptical about the value of BBCC after having participated in the training. They also reported a change in their counselling style and in the patients’ responses. The patient centred approach resulted in less resistance from patients to change and improved relationships between providers and patients. There were no discernible differences between the attitude and beliefs of low, medium and high scorers.

“*You don’t feel that you have to say by the end of the day, you made five patients stop smoking” (Doctor, ID: 2)*

*“If you put the responsibility over to the patient, I think it takes away a lot of frustration because in the end it’s the patient that needs to make the decision.” (Nurse, ID: 8)*

*“I never used to think that this was part of my job, but now I feel that although it’s going to be difficult where I work, I can do it” (Doctor, ID :6)*

The training not only changed their attitude towards their previous counselling habits, but also made them feel valued.

*“I definitely feel that I’m doing much more for the patient” (Doctor, ID: 11)*

*“They come back to say: ‘Sister I’m at this point’ (in terms of where they are in the process of change), and then I’m so proud of myself” (Nurse, ID :7)*

Although PCPs confidence improved, they experienced difficulties with some aspects of the new approach, for instance, assessing a patient’s readiness to change, and letting go of the “expert” role. Some PCPs said that they had difficulty when using scaling questions to rate the patient’s confidence and the importance of changing their behaviour. PCPs did not always feel it was appropriate to use this type of questions, and that the training was too prescriptive on how to ask this.

*“One thing that I still don’t like from this, is that assessment thing, but people still struggle to give me a number, like they really struggle to, and if I push them for a number, it feels that it becomes very mechanical” (Nurse, ID:8)*

*“It [*assessment step*] becomes a very mechanic exercise, more than actually getting down to what it actually was supposed to do” (Doctor, ID:12)*

#### Experiences of implementing the approach in clinical practice

According to some respondents it was easy for them to default to the “expert role” because they are immersed in a culture that fosters this approach. The organisational culture was quite controlling and appeared to evoke a similar culture in the consultation whereby practitioners tried to control patient choice and reduce their sense of autonomy:

*“It’s difficult for me to make that change, to let go, because you are not in control then. And I think sometimes we like to control our patients, it’s our job. ” (Nurse, ID: 8)*

*“You do follow your example set by your superiors, I suppose. And that does, it sets a different environment or a climate, of actually doing things like that. I mean, at the clinic, everything is very clinical at the moment, and not really much time for counselling and I really think it is a mind- set that I suppose comes from top to bottom.” (Doctor, ID: 2)*

The training changed PCP’s perceptions about some of the previously reported barriers to counselling, for instance, time constraints.

*“As doctors, we always, we do have limited time, but we like to hide behind that as an excuse as well, not to do behavioural counselling, or any counselling for that matter.” (Doctor, ID: 2)*

*“It takes a bit longer in the beginning, but as you progress it becomes easier.” (Nurse, ID: 5)*

*“Just by spending a little more time with her, we were able to identify what was her barrier to losing weight.” (Doctor, ID: 10)*

In contrast, time pressures were still a significant barrier for other respondents:

*“It’s also with the expectation of the clinic [and the patients themselves] that you need to get this done, and the patient sitting outside knocks on the door and says: Are you not finished yet?” (Nurse, ID: 8)*

Some of the other barriers that were still experienced were language issues, poor record keeping, understaffed facilities, lack of support within the facility, poor continuity of care, and lack of patient support materials:

“*Language yes, because some people only want to be addressed in their home language, which is not always English. My main barrier now is the third language. I don’t speak an African language, and a lot of people in the community need you to speak their language.” (Nurse, ID: 9)*

*“I don’t really get to see my patients again” (Doctor, ID: 4)*

*“Yes well, in my ideal world I would have all the beautiful pamphlets with me and all the tools that I need to actually do it which includes time and just the paperwork…. I’m working in different places and I don’t know what the resources are in the community” (Doctor, ID: 10)*

#### Experiences with the course materials in clinical practice

PCPs valued the training manual, and used it not only as a reference, but also to share information with patients during counselling. Some thought that translating the patient support leaflets into local languages would be helpful:

*“You don’t have to remember all of it [training manual], you can actually have the patient just look in the book as well. You can share it [patient education leaflet] with the patient, you can open the book and tell the patient, if you can read let’s go through this” (Nurse, ID: 7)*

*“It’s[patient education leaflet] only in English, not Xhosa or Afrikaans, you know? That was an issue for me.” (Doctor, ID: 12)*

#### Ideas about future training programmes

Although future training was viewed as necessary for both doctors and nurses, respondents felt that other categories of health care workers, such as community health workers and physiotherapists should be included, as they also deal with patients with these risk factors. Some thought the approach could even be used for other behaviour change such as adherence to medication

*“I think it’s for all health workers, because we all deal with patients, not only nurses, not only doctors, like all the categories.” (Nurse, ID: 7)*

*“So I wondered if it would be an interesting thing to look at in HIV Care which is now one of our chronic illnesses, as well. Adherence is a huge problem with/for them, so having some kind of structure to follow?” (Doctor, ID: 12)*

Training was viewed as helpful and necessary to up skill PCPs, although PCPs felt that in their current work environment there was limited time and few staff and that organisational support for future training for others was lacking:

*“In the work that we do, we are few people, and if there is an emergency and they call me, I have to leave everything, I wish we could be more, do you understand?” (Nurse, ID: 3)*

*“I think they probably have a set way, but if there’s a chance that they can, [train] I think they will be open to it, there must just be time available to train.” (Doctor, ID: 6).*

Training established PCPs, especially nurses, to change their current style of counselling was seen as challenging. Respondents recommended that training should be introduced at an earlier stage, because it is easier to start off with a patient centred approach, than to try to change old habits:

*“I don’t see that any doctors want to change what they have been taught.” (Doctor, ID: 10)*

*“This is something that should be part of the curriculum of the undergraduate nursing students; because it will help them to develop this manner of dealing with the patient, and not ending up with the bad ways and then trying to rectify that, but starting off with the correct method.” (Nurse, ID: 9)*

*“A lot of them are old school, so I think that it will be difficult to break down the barriers, and way of thinking, and attitude towards counselling.” (Doctor, ID: 4)*

## Discussion

The findings of this qualitative study are congruent with the findings of the quantitative evaluation, and demonstrated that in the short term, a once off training intervention can change PCP’s perception of the importance of delivering BBCC in the South African primary care context [13]. Training helped PCPs to recognize the worth of their possible contribution, and provided them with the necessary skills to perform BBCC, which increased their confidence in performing BBCC in actual practice.

It echoes other studies that have found that trained PCPs are less sceptical and feel more confident in their ability to deliver BBCC in clinical practice [18, 19]. BBCC training can change the approach of PCPs to delivering BBCC by changing their underlying values and beliefs.

However, although training enhances PCP’s perceived efficiency and capacity to provide this counselling, implementing it into every day practice in the long term remains challenging [18]. In this study, although PCP’s confidence in counselling improved, and some thought that time constraints could be overcome, they still reported that understaffing, lack of support from within the facility and poor continuity of care were barriers to counselling. Within this environment some found it challenging not to default back to the directive approach of counselling. It is clear that to incorporate BBCC into everyday care, a whole systems approach is needed, that involves the patient, provider, and service organisation at different levels [20, 21].

PCPs in the Cape Town public sector have been characterised by personal values of caring, respect, compassion and listening, all of which are well aligned with the approach to BBCC [21]. In other words BBCC could enable these personal values to find expression in the context of the consultation. The training programme therefore enabled PCPs to develop skills and professional behaviour that were well aligned with these values. However, although training can enhance this personal alignment, this is not sufficient to embed BBCC in every day practice. Another aspect of personal alignment is in helping staff to change their own unhealthy behaviours, and be good examples of a healthy lifestyle as this has been found to enhance performance in BBCC [18].

It is clear from the findings that there is a significant malalignment of personal and organisational values. Although the organisation espouses values of caring, competence, accountability, integrity, responsiveness and respect; the organisational culture is actually experienced as one of not sharing information, control, manipulation, blame, and power [21]. This organisational culture is not congruent with the patient-centred guiding style of BBCC and may provide an unsupportive and undermining environment [6]. Changing such organisational culture will require leadership transformation and a concerted effort to make the espoused values and culture a lived reality. Improving relationships trust and communication amongst the staff and management may be an important issue to support BBCC. It may be necessary to provide managers with the evidence for BBCC and to engage with them in a discussion about it [20–23].

In addition to creating a more supportive and committed organisational culture for BBCC it is necessary to ensure that the organisational practices and processes are also congruent with a patient-centred approach. For example it may be necessary to provide educational resources, to recognise and reward the provision of BBCC, and to commit to training all relevant staff in behaviour change counselling skills. Although there are many competing organizational demands in the daily operation of primary care centres, ongoing support for PCPs to offer BBCC should be prioritised [20]. Ultimately piloting BBCC in different clinics may be a step towards widespread implementation [18, 24, 25].

Nurses play a vital role in service delivery in primary care facilities and local and international findings demonstrate that nurses can be effective in providing BBCC [20]. However, a local culture of surveillance in primary care is dictated by bureaucracies in an attempt to ensure accountability [21, 26]. Task orientation is entrenched in nursing practice because it enables nurse managers to measure and to some extent control nurse’s performance and because it enables distance between the nurse and the patient. This system of discipline and scrutiny in which nurses are regulated, may be internalised and lead to a similar approach to patients, which inhibits the caring holistic approach that is integral to BBCC [26]. To create this caring culture in healthcare institutions, we may need a shift away from fragmentation of nurses work into tasks. Clinical governance, which focuses on measuring and improving the quality of care, should find ways of not just quantifying counselling in a tick-box approach, but of valuing and assessing the nature and quality of counselling.

Evidence has shown that although integration of a system of chronic care in primary care facilities with limited financial resources is feasible, weak national systems often make it difficult to implement and sustain these interventions [27, 28]. Although BBCC integrated into everyday routine primary care has been prioritized by the NDOH, this study demonstrates the gap between national aspirations and the realities of incorporating BBCC into everyday service delivery [3]. Patient centred care should be the central ambition of chronic care development strategies and policies that work towards outcomes that matter to patients, and not just to programmes or disease-orientated guidelines.

The findings of this study suggests that training should be incorporated early on, preferably into undergraduate curricula of PCPs for both nurses and doctors, to ensure that behaviour change counselling skills are embedded from the start. Existing PCPs could be offered training as part of continued professional development programmes. Internationally, the importance of incorporating BBCC training into curricula for PCPs, has been recognised as a future step in the struggle against NCDs [29–31].

This study forms part of a bigger project, the ichange4health programme, which has helped to further develop the materials and train trainers from Departments of Family Medicine and Primary Care throughout South Africa [6]. These trainers are now able to train medical students, general practitioners and other family physicians in their respective areas.

Training PCPs in behaviour change counselling skills can have broader application beyond the risky lifestyle behaviours associated with NCDs. For example consultations involving risky sexual behaviours, intimate partner violence and problems with adherence to chronic medication could benefit from such a skills set. Future research should look at developing a comprehensive approach to patient education and counselling that includes BBCC as one component.

One of the limitations of this study was the limited follow up period in clinical practice. If interviewees were interviewed at a later time, it could have led to different responses. Implementing and improving a new skill over time is unlikely in a culture where limited support and feedback on performance is available and therefore one might anticipate a gradual loss of motivation and positivity. Future research is intended to evaluate the effect of providing on going on-line support after training and evaluating retention of the approach to BBCC over a longer time period. As the he researcher had been primarily involved in training and interviewing the PCPs, there could have been obsequiousness bias in the responses given. Previous research conducted by the researcher on general practitioners’ poor management of overweight and/or obese patients, could have had a negative influence on her perception of the PCP’s efficacy in counselling [16]. However, the fact that the interview process, analysis and interpretation were, supervised by the other co-authors would have mitigated this influence.

## Conclusion

This study showed that an eight hour training intervention for PCPs on BBCC changed PCPs perception of its importance, their ability to offer BBCC, and their confidence to overcome certain barriers to implementation. Nevertheless significant barriers to implementation remained such as language, time constraints, poor continuity of care, an unsupportive organisational culture, and lack of educational resources. In future, training should be incorporated into nurses, doctors and other primary care provider’s basic curricula in order to provide a stronger foundation. However, to fully embed and sustain BBCC a whole system approach may be needed and not just a commitment to training.

## Abbreviations

BBCC: Brief behaviour change counselling
LMIC: Low and middle income countries
NDOH: National department of health
NCD’s: Non communicable diseases
PCP: Primary care providers
